# Structural insights into the molecular mechanism of high-level ceftazidime–avibactam resistance conferred by CMY-185

**DOI:** 10.1128/mbio.02874-23

**Published:** 2024-01-05

**Authors:** Akito Kawai, William C. Shropshire, Masahiro Suzuki, Jovan Borjan, Samuel L. Aitken, William C. Bachman, Christi L. McElheny, Micah M. Bhatti, Ryan K. Shields, Samuel A. Shelburne, Yohei Doi

**Affiliations:** 1 Department of Microbiology, Fujita Health University School of Medicine, Toyoake, Aichi, Japan; 2 Center for Infectious Disease Research, Fujita Health University, Toyoake, Aichi, Japan; 3 Department of Infectious Diseases, Infection Control, and Employee Health, The University of Texas MD Anderson Cancer Center, Houston, Texas, USA; 4 Division of Pharmacy, The University of Texas MD Anderson Cancer Center, Houston, Texas, USA; 5 Division of Infectious Diseases, University of Pittsburgh School of Medicine, Pittsburgh, Pennsylvania, USA; 6 Division of Pathology/Lab Medicine, Department of Laboratory Medicine, The University of Texas MD Anderson Cancer Center, Houston, Texas, USA; 7 Department of Infectious Diseases, Fujita Health University School of Medicine, Toyoake, Aichi, Japan; IBR (CONICET) University of Rosario, Rosario, Santa Fe, Argentina

**Keywords:** ceftazidime, avibactam, cefiderocol, beta-lactamase, CMY-2 variant

## Abstract

β-Lactamases can accumulate stepwise mutations that increase their resistance profiles to the latest β-lactam agents. CMY-185 is a CMY-2-like β-lactamase and was identified in an *Escherichia coli* clinical strain isolated from a patient who underwent treatment with ceftazidime-avibactam. CMY-185, possessing four amino acid substitutions of A114E, Q120K, V211S, and N346Y relative to CMY-2, confers high-level ceftazidime-avibactam resistance, and accumulation of the substitutions incrementally enhances the level of resistance to this agent. However, the functional role of each substitution and their interplay in enabling ceftazidime-avibactam resistance remains unknown. Through biochemical and structural analysis, we present the molecular basis for the enhanced ceftazidime hydrolysis and impaired avibactam inhibition conferred by CMY-185. The substituted Y346 residue is a major driver of the functional evolution as it rejects primary avibactam binding due to the steric hindrance and augments oxyimino-cephalosporin hydrolysis through a drastic structural change, rotating the side chain of Y346 and then disrupting the H-10 helix structure. The other substituted residues E114 and K120 incrementally contribute to rejection of avibactam inhibition, while S211 stimulates the turnover rate of the oxyimino-cephalosporin hydrolysis. These findings indicate that the N346Y substitution is capable of simultaneously expanding the spectrum of activity against some of the latest β-lactam agents with altered bulky side chains and rejecting the binding of β-lactamase inhibitors. However, substitution of additional residues may be required for CMY enzymes to achieve enhanced affinity or turnover rate of the β-lactam agents leading to clinically relevant levels of resistance.

**IMPORTANCE:**

Ceftazidime-avibactam has a broad spectrum of activity against multidrug-resistant Gram-negative bacteria including carbapenem-resistant *Enterobacterales* including strains with or without production of serine carbapenemases. After its launch, emergence of ceftazidime-avibactam-resistant strains that produce mutated β-lactamases capable of efficiently hydrolyzing ceftazidime or impairing avibactam inhibition are increasingly reported. Furthermore, cross-resistance towards cefiderocol, the latest cephalosporin in clinical use, has been observed in some instances. Here, we clearly demonstrate the functional role of the substituted residues in CMY-185, a four amino-acid variant of CMY-2 identified in a patient treated with ceftazidime-avibactam, for high-level resistance to this agent and low-level resistance to cefiderocol. These findings provide structural insights into how β-lactamases may incrementally alter their structures to escape multiple advanced β-lactam agents.

## INTRODUCTION

Multidrug resistance (MDR) in Gram-negative bacteria is a growing global public threat, of which resistance to β-lactam agents due to the production of β-lactamases is a major component. Avibactam is a potent inhibitor of the classes A, C, and some D β-lactamases, and is combined with the third-generation cephalosporin ceftazidime for the treatment of MDR Gram-negative bacterial infection. Ceftazidime-avibactam has robust *in vitro* activity against *Klebsiella pneumoniae* carbapenemase (KPC)-producing *Enterobacterales*, and favorable clinical outcomes have been reported for the patients infected with carbapenem-resistant pathogens and treated with this agent (1). Ceftazidime-avibactam is also active against non-carbapenemase-producing carbapenem-resistant *Enterobacterales* that produce class C β-lactamases, also known as AmpC β-lactamases, or extended-spectrum β-lactamases (ESBLs) (2). However, after the launch of ceftazidime-avibactam, emergence of ceftazidime-avibactam-resistant strains that produce mutant β-lactamases capable of efficiently hydrolyzing ceftazidime or resisting avibactam inhibition are increasingly reported (3–6).

CMY-2 is the most common plasmid-mediated AmpC β-lactamase and is inhibited by avibactam (7). CMY-185, a four amino acid variant of CMY-2, was identified in an *Escherichia coli* clinical strain isolated from a patient who underwent treatment with ceftazidime-avibactam. *bla*_CMY-185_ confers high-level ceftazidime-avibactam resistance with an MIC of 32 mg/L (8). CMY-185 possesses four amino acid substitutions of A114E, Q120K, V211S, and N346Y in comparison to the CMY-2 sequence. We previously reported the antibiotic susceptibility profiles of *E. coli* laboratory strain TOP10 producing the CMY-2 variants for every combination of the four amino acid substitutions A114E, Q120K, V211S, and N346Y observed in CMY-185 (8). These results suggested that the N346Y substitution in CMY-185 appeared to be a major driver of ceftazidime-avibactam resistance, but that additional substitutions were required for high-level resistance (8). The residues at position 346 among AmpC β-lactamases are either asparagine or isoleucine (9, 10). The N346 residue of AmpC β-lactamases directly interacts with the C4 carboxyl group on the dihydrothiazine ring of cephalosporin and the sulfate group of avibactam (11–15). We hypothesized that the N346Y substitution of CMY-185 disrupts these interactions, sterically clashes with the substrates, and reduces the binding affinity of both ceftazidime and avibactam. As a result, a strain producing the enzyme containing the N346Y substitution would be susceptible to ceftazidime due to a functional tradeoff between resistance to ceftazidime and avibactam. However, previous biochemical studies of AmpC β-lactamases possessing the N346Y substitution revealed that these enzymes retained the hydrolytic efficiency against cephalosporins including ceftazidime but had impaired avibactam inhibition (9). These findings prompted us to investigate the molecular mechanism underlying ceftazidime-avibactam resistance conferred by CMY-185 and reveal the functional role of each observed substitution, namely A114E, Q120K, V211S, and N346Y. Here, we report the biochemical and structural characterization of CMY-185 as well as the CMY-2 variants harboring each substitution.

## RESULTS

### Kinetic parameters of the CMY-2 variants

We tested the enzymes, CMY-2, two single mutants V211S (CMY-42) and N346Y, three double mutants A114E_N346Y, Q120K_N346Y, and V211S_N346Y, and the quadruplet mutant CMY-185 to elucidate the impact on the kinetic properties resulting from the four amino acid substitutions present in CMY-185 compared with CMY-2 (8). The steady-state kinetic parameters *k*_cat_ and *K*_m_ (or *K*_i app_) for nitrocefin, cephalothin, ceftazidime, and cefiderocol and the inhibition parameters *K*_i app_, *k*_2_/*K*, *k*_−2_, and *k*_off_ for avibactam are summarized in Table 1. During the hydrolysis of nitrocefin, we observed the reduction of the initial velocity at concentrations above 50 µM particularly for the CMY-2 variants harboring the N346Y substitution. As this could have represented substrate inhibition, we then determined the kinetics parameters for nitrocefin using the Haldane equation (16). For ceftazidime or cefiderocol, no apparent hydrolysis was observed by CMY-2, N346Y, A114E_N346Y, and Q120K_N346Y (the latter only for cefiderocol) at the substrate concentration of 100 µM with 100–200 nM of the enzyme; thus, we determined each *K*_i app_ through the hydrolysis of reporter substrate cephalothin according to a previous report (17). In addition, during the hydrolysis of cefiderocol by CMY-185, the initial velocities at a concentration range of 3.7–59.4 µM were similar. These results indicated that the value of *K*_m_ is very low and that it is difficult to determine the initial velocity at lower concentrations of cefiderocol than what we measured due to the undetectable absorbance changes caused by the hydrolysis of cefiderocol. Cefiderocol is a poorly hydrolyzed substrate for CMY-185 compared to cephalothin, thus we determined *K*_i app_ for cefiderocol of CMY-185 through the hydrolysis of reporter substrate cephalothin, and used it, which is assumed to be comparable to *K*_m_, to calculate the other kinetic parameters (18, 19).

**TABLE 1:** Kinetic and inhibition parameters of the CMY-2 variants^*c*^

		CMY-2 variants						
Substrate	Kinetics parameters	Without mutation (CMY-2)	V211S (CMY-42)	N346Y	A114E_N346Y	Q120K_N346Y	V211S_N346Y	A114E_Q120K_ V211S_N346Y (CMY-185)
Nitrocefin								
	*k*_cat_ (s^−1^)	2.4 × 10^2^ ± 0.5 × 10^2^	3.2 × 10^2^ ± 1.02 × 10^2^	61 ± 5	57 ± 10	1.2 × 10^2^ ± 0.9 × 10^2^	85 ± 18	17 ± 9
	*K*_m_ (µM)	17 ± 5	29 ± 12	25 ± 3	16 ± 4	50 ± 40	35 ± 10	39 ± 24
	*K*_i_ (µM)	1.6 × 10^2^ ± 1.0 × 10^2^	1.3 × 10^2^ ± 0.7 × 10^2^	87 ± 25	68 ± 37	14 ± 12	1.0 × 10^2^ ± 0.5 × 10^2^	18 ± 13
	*k*_cat_/*K*_m_ (µM^−1^ s^−1^)	14 ± 5	11 ± 5	2.5 ± 0.4	3.6 ± 1.2	2.4 ± 2.5	2.4 ± 0.9	0.4 ± 0.4
								
Cephalothin								
	*k*_cat_ (s^−1^)	54 ± 0.8	83 ± 7	18 ± 7	3.3 ± 0.1	42 ± 5	9.6 ± 0.2	14 ± 1
	*K*_m_ (µM)	4.1 ± 0.3	7.5 ± 1.1	3.9 ± 0.1	1.4 ± 0.1	18 ± 3	4.4 ± 0.3	19 ± 1
	*k*_cat_/*K*_m_ (µM^−1^ s^−1^)	13 ± 1	11 ± 2	4.7 ± 1.8	2.3 ± 0.2	2.4 ± 0.5	2.2 ± 0.2	0.8 ± 0.1
								
Ceftazidime								
	*k*_cat_ (s^−1^)	N.D.	1.5 ± 0.2	N.D.	N.D.	7.7 × 10^−2^ ± 0.3 × 10^−2^	0.3 ± 0.0	0.1 ± 0.0
	*K*_m_ (µM)	N.M.	74 ± 10	N.M.	N.M.	5.3 ± 0.5	37 ± 2	2.2 ± 0.3
	*K*_i app_ (µM)	0.7 ± 0.1	N.M.	0.7 ± 0.2	0.3 ± 0.0	N.M.	N.M.	N.M.
	*k*_cat_/*K*_m_ (µM^−1^ s^−1^)	N.C.	2.0 × 10^−2^ ± 0.3 × 10^−2^	N.C.	N.C.	1.5 × 10^−2^ ± 0.2 × 10^−2^	9.0 × 10^−3^ ± 0.7 × 10^−3^	5.3 × 10^−2^ ± 0.7 × 10^−2^
								
Cefiderocol								
	*k*_cat_ (s^−1^)	N.D.	3.4 × 10^−2^ ± 0.2 × 10^−2^	N.D.	N.D.	N.D.	4.9 × 10^−2^ ± 0.3 × 10^−2^	6.9 × 10^−3^ ± 0.4 ×10^−3 *a*^
	*K*_m_ (µM)	N.M.	68 ± 11	N.M.	N.M.	N.M.	6.9 ± 1.5	N.M.
	*K*_i app_ (µM)	8.0 ± 1.3	N.M.	0.7 ± 0.0	0.4 ± 0.0	0.4 ± 0.0	N.M.	0.2 ± 0.0
	*k*_cat_/*K*_m_ (µM^−1^ s^−1^)	N.C.	4.9 × 10^−4^ ± 0.9 × 10^−4^	N.C.	N.C.	N.C.	7.1 × 10^−3^ ± 1.6 × 10^−3^	3.0 × 10^−2^ ± 0.2 × 10^−2 *b*^
								
Avibactam								
	*K*_i app_ (µM)	6.3 × 10^−2^ ± 1.2 × 10^−2^	3.4 × 10^−2^ ± 0.6 × 10^−2^	41 ± 6	80 ± 9	5.9 × 10^2^ ± 0.2 × 10^2^	14 ± 4	1.3 × 10^3^ ± 0.1 × 10^3^
	*k*_2_/*K* (M^−1^ s^−1^)	2.8 × 10^4^ ± 0.1 × 10^4^	2.3 × 10^4^ ± 0.1 × 10^4^	59 ± 6	41 ± 0.4	32 ± 0.4	66 ± 6	7.4 ± 0.3
	*k*_−2_ (s^−1^)	1.2 × 10^−4^ ± 0.2 × 10^−4^	1.5 × 10^−4^ ± 0.2 × 10^−4^	1 × 10^−5^ ± 16 × 10^−5^	1.1 × 10^−4^ ± 0.0 × 10^−4^	2.0 × 10^−3^ ± 0.1 × 10^−3^	4.4 × 10^−4^ ± 2.1 × 10^−4^	7.0 × 10^−4^ ± 0.5 × 10^−4^
	*k*_off_ (s^−1^)	7.3 × 10^−5^ ± 0.0 × 10^−5^	6.0 × 10^−5^ ± 0.0 × 10^−5^	2.6 × 10^−6^ ± 0.0 × 10^−6^	9.0 × 10^−7^ ± 0.0 × 10^−7^	4.0 × 10^−6^ ± 0.0 ×10^−6^	2.4 × 10^−6^ ± 0.0 × 10^−6^	4.8 × 10^−7^ ± 0.0 × 10^−7^

^*a*^ Due to the small *K*_m_ value of CMY-185 for cefiderocol, the *k*_cat_ value was determined with the measured velocities at the steady state (cefiderocol concentration range: 3.7–59.4 µM) and the *K*_i app_ value assumed to *K*_m_.

^*b*^ The *k*_cat_/*K*_i app_ value is shown.

^*c*^ The values are the mean ± standard error of three independent measurements. N.M. = not measured; N.D. = not detected; N.C. = not calculated.

### V211S of CMY-2 variants augments the hydrolysis of ceftazidime and cefiderocol

The kinetics parameters for nitrocefin and cephalothin showed that hydrolytic efficiency of CMY-2 was at least threefold higher than that of the CMY-2 variants except for those with V211S, and the hydrolytic efficiency tended to decline as the mutations accumulated. The *k*_cat_ and *K*_m_ values for V211S were higher than those for CMY-2, resulting in comparable hydrolytic efficiency of nitrocefin and cephalothin between the two enzymes. For ceftazidime, the *k*_cat_ and *K*_m_ values of V211S and V211S_N346Y were higher than those of Q120K_N346Y and CMY-185. The *K*_i app_ values of CMY-2, N346Y, and A114E_N346Y were 0.3–0.7 µM and are an order magnitude lower than those for which the *K*_m_ values could be determined. For AmpC β-lactamase, deacylation is considered to be a rate-limiting step for the third-generation cephalosporins (20, 21). Thus, the *K*_i app_ values of CMY-2, N346Y, and A114E_N346Y may be lower than actual *K*_m_ value. For cefiderocol, we could determine the kinetic parameters *k*_cat_ and *K*_m_ on the CMY-2 variants harboring the V211S substitution. The *k*_cat_ and *K*_m_ values for V211S were (3.4 ± 0.2) × 10^−2^ s^−1^ and 68 ± 11 µM, respectively. The *K*_m_ value of V211S_N346Y was 10-fold lower than that of V211S, while the *k*_cat_ values were comparable. The *K*_m_ (*K*_i app_) value of CMY-185 was further lower, while the *k*_cat_ value was also reduced. Comparing cefiderocol with ceftazidime, the *K*_i app_ value of CMY-2 was 10-fold higher, while the values of *K*_i app_ of N346Y and A114E_N346Y were comparable. These results indicated that the V211S substitution conferred superior turnover rates for most cephalosporins. The roles of the other substitutions A114E, Q120K, and N346Y in the hydrolysis of the cephalosporins are complex. In CMY-185, these substitutions appeared to enhance the hydrolysis of oxyimino-cephalosporins ceftazidime and cefiderocol due to the maintenance of the binding affinity. However, against the classical cephalosporins nitrocefin and cephalothin, it exhibited lower turnover rate or binding affinity, resulting in inferior hydrolysis compared to CMY-2.

### The CMY-2 variants harboring N346Y and the Q120K_N346Y combination impair avibactam inhibition

The inhibitory constants *K*_i app_ of avibactam for CMY-2 and V211S were 62 ± 12 and 34 ± 6 nM, respectively. Compared to these enzymes, N346Y showed a 1,000-fold higher *K*_i app_ value, and Q120K_N346Y and CMY-185 showed 10,000- and 20,000-fold higher values, respectively. These results indicated that the N346Y substitution abolished avibactam inhibition, and the additional Q120K substitution contributed to enhanced resistance to avibactam inhibition. Avibactam is a covalent reversible inhibitor, and it binds to the enzyme, carbamoylates the active serine residue (S64 in CMY-185), then departs the enzyme by decarbamoylation through hydrolysis or intramolecular ring closure of avibactam, resulting in the release of regenerated avibactam (22). To reveal the mechanism underlying the impairment of avibactam inhibition, we determined the second-order carbamoylation rate constant *k*_2_/*K* and the decarbamoylation rate constants *k*_−2_ and *k*_off_ for each enzyme (23). These results showed substantial reduction (~1,000-fold) in *k*_2_/*K* values for the CMY-2 variants harboring the N346Y substitution in comparison to those for CMY-2 or V211S, while the *k*_−2_ values were comparable with each other and the *k*_off_ values were reduced as the substitutions were accumulated. The effect of the additional Q120K substitution was more complex in that the *k*_2_/*K*, *k*_−2_, and *k*_off_ values were comparable with those of the CMY-2 variants harboring the N346Y substitution. However, the ratios of *k*_−2_ to *k*_2_/*K* of Q120K_N346Y and CMY-185 were slightly higher (~10-fold) than those for the CMY-2 variants harboring the N346Y substitution suggesting that, compared with the others, Q120K_N346Y and CMY-185 may release the avibactam molecule faster, before they are carbamoylated by avibactam.

### The N346Y substitution induces a drastic structural change of the R2 loop in CMY-185 when ceftazidime is bound

**Fig 1:**
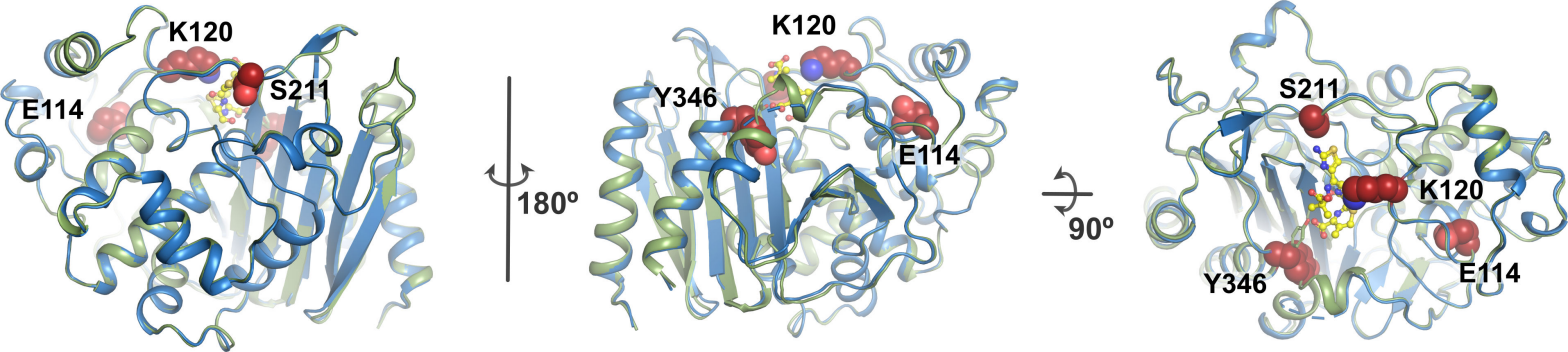
Overall structure of CMY-185. Crystal structures of CMY-185 are shown as cartoon representations colored green for the free form and blue for the complex with ceftazidime. The substituted residues E114, K120, S211, and Y346 are shown as CPK representations colored red. The ceftazidime molecule is shown as a ball-and-stick representation colored yellow.

To obtain further structural insights on CMY-185, we determined the crystal structures of the CMY-185 free form and its complex with ceftazidime at the resolution of 1.35 and 2.40 Å, respectively. We prepared the crystals of the CMY-185-ceftazidime complex by soaking a crystal of the CMY-185 free-form in mother liquor containing 100 mM ceftazidime for 4 h. Data collection and refinement statistics are summarized in Table 2. To date, crystal structures of CMY-2 (PDB ID 1ZC2) and CMY-136 (PDB ID 6G9T) (24), a CMY-2 variant harboring the Y221H substitution, have been deposited in PDB database among the CMY-2-like β-lactamases. Structural comparison with these enzymes showed that the CMY-185 free-form structure is quite similar to the structure of CMY-2 and CMY-136 with the root-mean-square deviation (RMSD) values of 0.45 and 0.52 Å, respectively. The substituted residues K120, S211, and Y346 in CMY-185 are located adjacent to the substrate binding site (Fig. 1). The residues S211 and Y346 form hydrogen bonds with residues E61 and S318, respectively, while the side chain of K120 is exposed to the bulk solvent. The substituted residue E114 is located at the inner molecule, and it forms hydrogen bonds with the main chain nitrogen atoms of G116 and L117. The effect of the A114E substitution to the overall structure appears to be minimal, resulting in the loop structure around the P118 residue pushed up ~1 Å away.

**TABLE 2:** Data collection and structure refinement statistics

Data set	Free form	Ceftazidime complex
Data collection		
Source	Photon Factory BL-17A	Photon Factory BL-17A
Wavelength (Å)	0.9800	0.9800
Space group	*P*2_1_2_1_2_1_	*P*2_1_2_1_2_1_
Unit-cell parameters		
Length (Å)	*a* = 79.7, *b* = 89.5, *c* = 104.3,	*a* = 81.1, *b* = 89.3, *c* = 104.8,
Resolution range (Å)	45.1–1.35 (1.43–1.35)	45.2–2.40 (2.55–2.40)
No. of observed reflections	2,207,927 (333,504)	410,355 (57,630)
No. of unique reflections	163,856 (26,083)	30,373 (4,823)
Multiplicity	13.5 (12.8)	13.5 (11.9)
Completeness (%)	99.8 (99.0)	99.9 (99.4)
*R*_merge_ (%)^*a*^	8.1 (88.8)	13.6 (87.8)
〈*I*/σ(*I*)〉	17.42 (2.72)	15.96 (2.96)
Refinement		
Resolution (Å)	39.1–1.35 (1.37–1.35)	45.2–2.40 (2.48–2.40)
Reflection used	163,761 (5,165)	30,319 (2,700)
*R*_work_ (%)^*b*^	13.9 (22.0)	18.6 (24.2)
*R*_free_ (%)^*c*^	17.7 (25.1)	23.0 (30.6)
No. of non-hydrogen atoms	7,055	5,740
Protein	5,965	5,498
Ligands	10	62
Solvent	1,080	180
RMSD from ideality		
Bond length (Å)	0.008	0.005
Bond angle (°)	0.940	0.742
Average B-factor	20.4	43.3
Protein	18.1	43.4
Ligands	28.2	44.0
Solvent	33.0	41.3
Ramachandran plot		
Favored region (%)	98.46	98.43
Allowed region (%)	1.54	1.57
Outlier region (%)	0.00	0.00
Clashscore	2.46	3.19
PDB ID	8JB7	8JB8

^*a*^ *R*_merge_ = 100 × ∑_*hkl*_ ∑_*i*_ | *I_i_(hkl)* − 〈*I(hkl)*〉 | / Σ*_hkl_* Σ*_i_ I_i_(hkl),* where 〈*I(hkl)*〉 is the mean value of *I(hkl)*.

^*b*^ *R*_work_ = 100 × ∑_*hkl*_| |*F*_*o*_ *| – |F_c_ | | /* ∑*_hkl_ |F_o_|* where *F_o_* and *F_c_* the observed and calculated structure factors, respectively.

^*c*^ *R*_free_ is calculated as for *R*_work_, but for the test set comprising 5% reflections not used in refinement.

**Fig 2:**
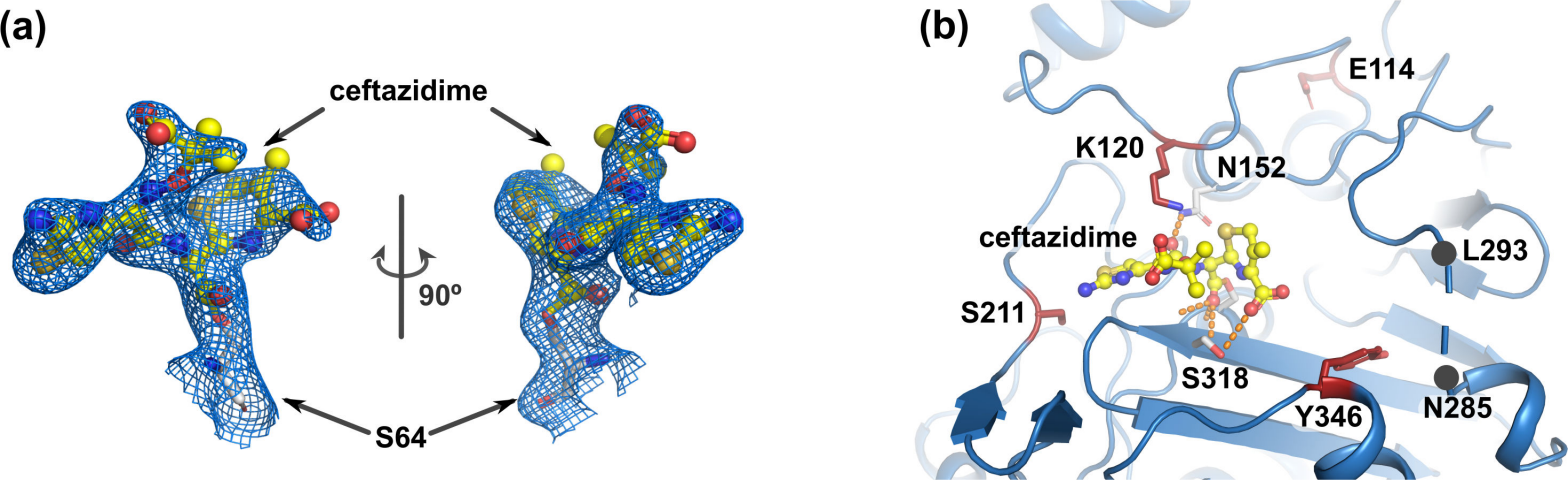
Ceftazidime binding site. The ceftazidime molecule is shown as a ball-and-stick representation colored yellow. (a) The S64 residue is shown as a white stick representation. 2m*F*_o_-D*F*_c_ map is shown as blue mesh contoured 1σ. (b) Hydrogen bonds are shown as orange dashed lines. Disordered residues are shown as blue dashed lines and the positions of N285 and L293 are marked as black dots.

**Fig 3:**
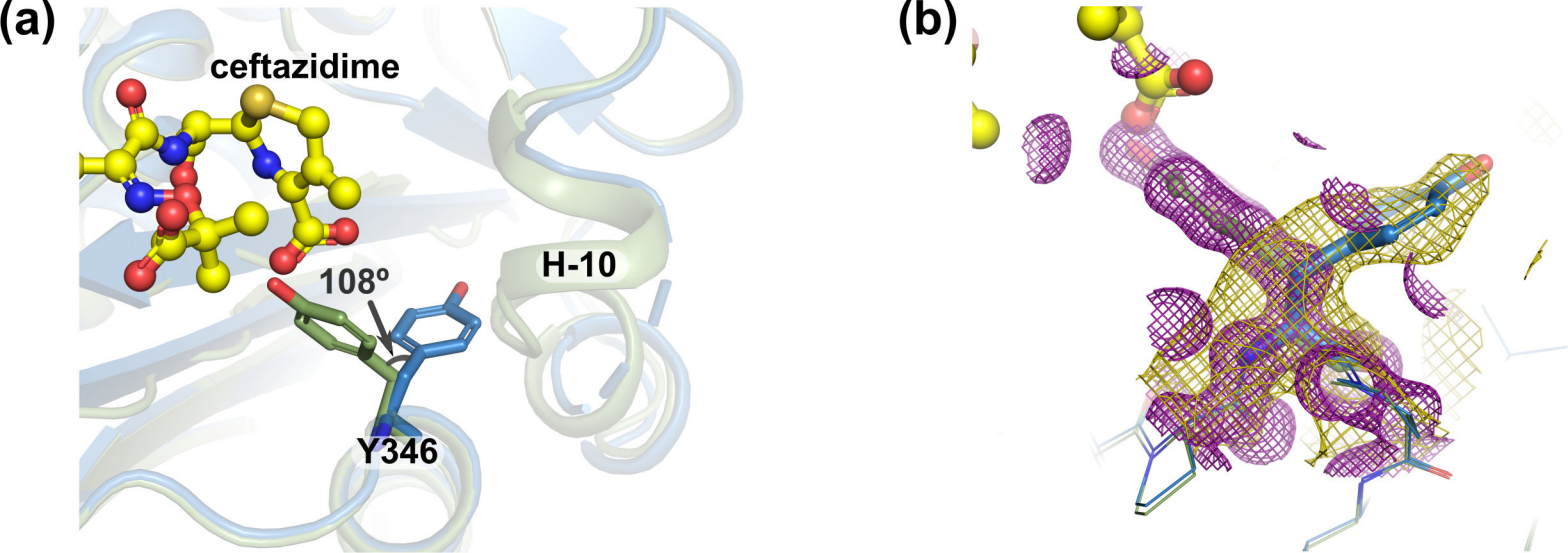
Structure comparison of the Y346 residue. (a) Structures of CMY-185 are colored green for the free form and blue for the complex with ceftazidime. (b) 2m*F*_o_-D*F*_c_ map is shown as purple (free form) and gold (complex) mesh contoured 1.25σ. The maps are drawn within 3 Å radius of each Y346 residue.

Crystal structure of the CMY-185-ceftazidime complex showed an acyl-enzyme intermediate where the S64 catalytic residue is covalently bound to the C8 atom of ceftazidime, and its β-lactam structure is cleaved (Fig. 3a). In contrast to the free form, crystal structure of the CMY-185-ceftazidime complex revealed a drastic structural change of the R2 loop in that the H-10 helix was disrupted and disordered at residues N285–L293 (Fig. 2). In comparing the crystal structures of the CMY-185 free form and its complex, the C4 carboxyl group on the dihydrothiazine ring of ceftazidime occupied the position of the side chain of Y346 observed in the free form. The side chain of Y346 in the complex in turn was rotated 108° toward the H-10 helix and positioned to clash with the S287 residue, and this appeared to cause the disruption of the H-10 helix structure (Fig. 3b). We did not observe overall structural changes except for the R2 loop with the RMSD value of 0.68 Å, and the positions of the substituted residues E114, K120, and S211 were almost identical. Structure comparisons of the CMY-185-ceftazidime complex with the other class C β-lactamase AmpC*^E. coli^* (PDB ID 1IEL) (11) and AmpC^Ent385^ (PDB ID 6LC9) (15) complex with ceftazidime showed that the binding position of the ceftazidime molecule was identical, and the hydrogen bond between Q120 and the amide group at the R1 side chain of ceftazidime disappeared by the substitutions, while S318 formed a hydrogen bond with the C4 carboxyl group of ceftazidime instead of N346 (Fig. 2).

### The substituted residues E114, K120, and S211 form stable hydrogen bonds

**Fig 4:**
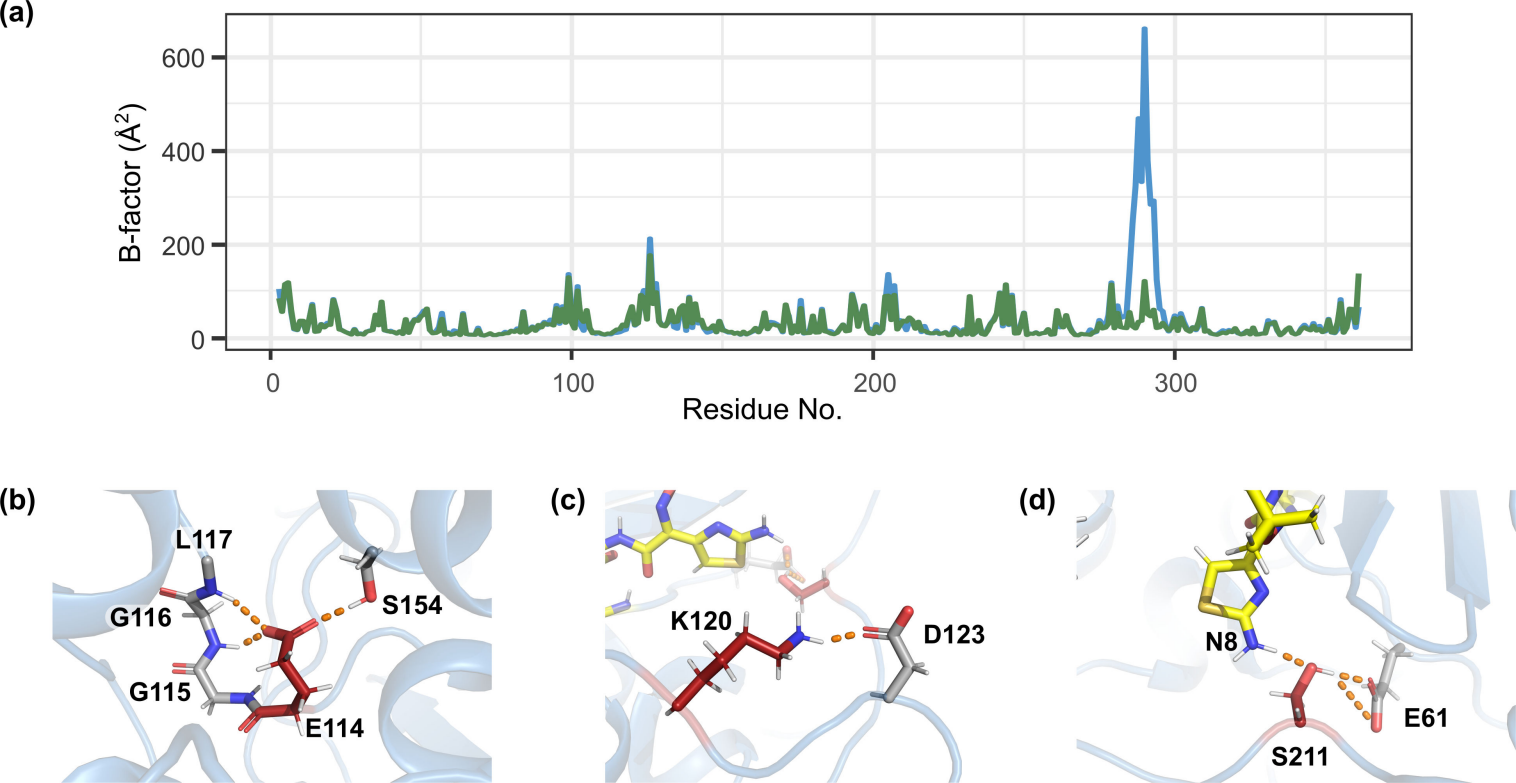
Molecular dynamics simulation for the CMY-2 and CMY-185 complexes with ceftazidime. (a) B factors profiles of the CMY-2 (green) and the CMY-185 (blue) complexes with ceftazidime. (b–d) Close-up view of the hydrogen bond or salt bridge formation of E114 (b), K120 (c), and S211 (d). The hydrogen bonds or salt bridge were shown as orange dashed lines.

Crystal structures of CMY-185 revealed that the N346Y substitution induced the R2 loop structure change due to the complex formation with ceftazidime. To elucidate further characteristics and potential effect of the other substituted residues on CMY-185, we examined the molecular behaviors of the CMY-185-ceftazidime complex using molecular dynamics simulations. Five runs of 100 ns molecular dynamics simulations were performed for the free form and the acyl complex with ceftazidime each of CMY-2 and CMY-185 at 300 K. The RMSD analysis indicated that the systems for both CMY-2 and CMY-185 reached equilibrium within 1 ns of the simulation (Fig. S1) and a stable conformation over the simulations, with mean RMSD values of 1.12 Å for the free form and 1.09 Å for the complex (Fig. S2a and S2b). Compared to CMY-2, CMY-185 showed slightly greater fluctuation, with mean RMSD values of 1.55 Å for the free form and 1.58 Å for the complex (Fig. S2c and S2d). Both CMY-2 and CMY-185 exhibited similar overall fluctuations between the free form and the complex (Fig. S1). Fig. 4a shows the overall profiles of the temperature factors for each residue. Compared to the CMY-2-ceftazidime complex, the CMY-185-ceftazidime complex structure showed more vibrations in the residues I284–L293 on the R2 loop, whereas the overall vibrations outside the R2 loop were similar. The residues I284–L293 almost correspond to the disordered residues in the crystal structure of the CMY-185-ceftazidime complex, suggesting that, in the crystal, the fragile structure was entirely disrupted by the complex formation, without the influence of crystal packing. Molecular dynamics simulation of the CMY-185-ceftazidime complex showed that the substituted residues formed stable hydrogen bonds or salt bridges. The side chain of E114 interacted with the side chain of S154 in addition to the main chain nitrogen atoms of G116 and L117 (Fig. 4b; Fig. S2). The side chain of K120 formed a salt bridge with the side chain of D123 (Fig. 4b; Fig. S3), while the side chain of S211 maintained a hydrogen bond with the side chain of E61 (Fig. 4c; Fig. S4).

## DISCUSSION

The N346 residue of typical AmpC β-lactamases directly interacts with cephalosporins and avibactam. Focusing on the disruption of the interaction, the N346Y substitution in the AmpC β-lactamases appears to confer functional tradeoff between resistance to cephalosporins and avibactam. However, in fact, this functional tradeoff is not always realized (8). Our structural studies presented here clearly demonstrate that the ceftazidime molecule is bound to CMY-185, and that this in turn leads to the drastic structural change of CMY-185 to accommodate the ceftazidime binding (Fig. 1 to 3). In addition, the kinetics studies indicated that, compared to CMY-2 or its V211S substitution mutant, the CMY-2 variants harboring the N346Y substitution had lower *k*_2_/*K* values (Table 1) and rejected the primary binding of avibactam like the other AmpC β-lactamases harboring the N346Y substitution (9). These results suggest that the sulfate group of avibactam is essential for its binding to the AmpC β-lactamases, while the cephalosporins are recognized through the overall interaction. This observation is supported by the number of hydrogen bonds between a typical AmpC β-lactamase and avibactam. The sulfate group of avibactam is recognized by three residues through ~5 hydrogen bonds, while carbamoyl group located at the opposite side of the sulfate group is recognized by the residues Q120 and N152 through two hydrogen bonds (12–15, 25). Moreover, in CMY-185, the Q120 residue is substituted to lysine, and the *K*_i app_ value of Q120K_N346Y is 10-fold higher compared to that of N346Y (Table 1). When the positions of nitrogen and oxygen atoms of the carbamoyl group of avibactam and the side chain of N152 were altered, the K120 residue in CMY-185 appeared to form a hydrogen bond with the oxygen atom of the carbamoyl group. However, the N152 residue also formed a hydrogen bond with the K67 residue conserved among the AmpC β-lactamases and could not rotate spontaneously. In addition, the molecular dynamics simulation indicated that the K120 residue formed a stable salt bridge with the D123 residue (Fig. 4c; Fig. S3), suggesting that the K120 residue was not primarily directed to interact with avibactam. These findings explain why the Q120K substitution combined with N346Y substitution enhances resistance to the avibactam inhibition.

The A114E substitution had a smaller effect on the enzyme reaction compared to the other substitutions. The kinetics studies of the CMY-2 variants harboring the A114E substitution showed that the values of *K*_m_ or *K*_i app_ for the cephalosporins measured in this study were generally lower than the other enzymes. On the other hand, the *K*_i app_ value for avibactam was higher than those of N346Y and V211S_N346Y, indicating that the A114E substitution affected the avibactam binding to the enzyme. Structural studies indicated that the A114E substitution did not alter the overall structure, and it instead seemed to enhance the intramolecular interaction through the hydrogen bond formation compared to alanine. We could define the phenotype of the CMY-2 variants harboring the A114E substitution, but the functional significance of the A114E substitution remains unclear.

**Fig 5:**
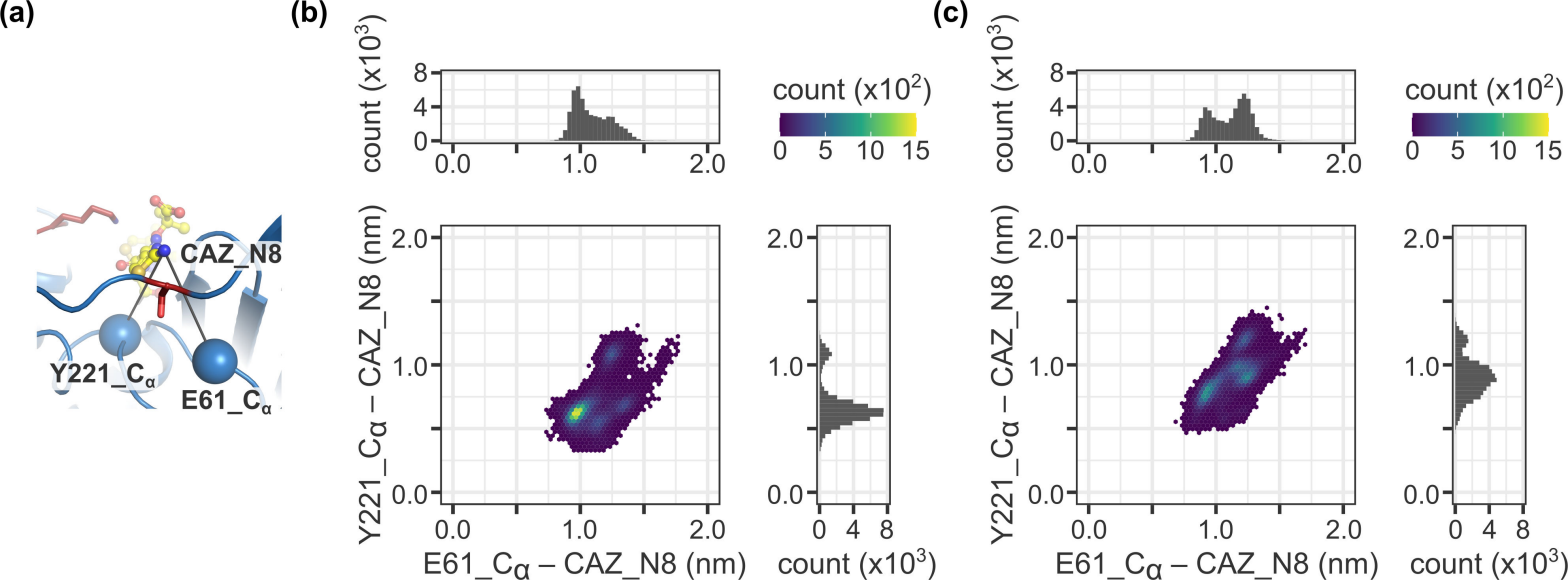
Fluctuations of the ceftazidime molecule bound to CMY-2 and CMY-185. (a) The C_α_ atoms for E61 and Y221 are shown as blue spheres. (**b, **c) 2D histogram for the distance distribution between the N8 atom of ceftazidime (CAZ_N8) and the C_α_ atoms of E61 (E61_C_α_) or Y221 (Y221_C_α_). Each point represents a snapshot from the molecular dynamics simulations, colored according to its counts shown in the color bar on the top right corner. (b) CMY-2. (c) CMY-185.

**Fig 6:**
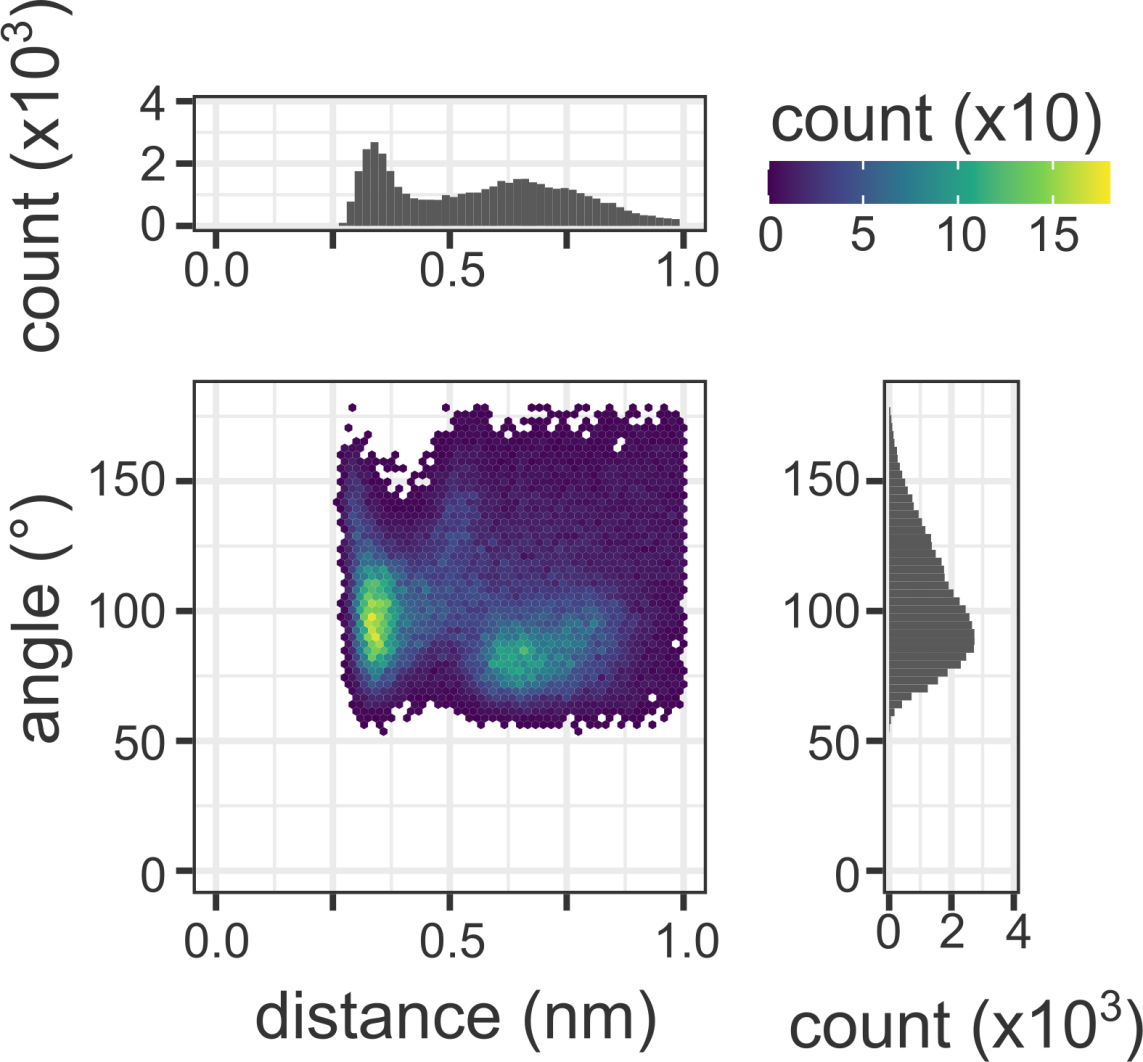
Interaction between S211 and the N8 atom of ceftazidime. 2D histogram for the distribution of the distance and angle between the O_γ_ atom of S211 and the N8 atom of ceftazidime. “Distance” axis indicates the distance between the O_γ_ atom of S211 and the N8 atom of ceftazidime. “Angle” axis indicates the angle between the atoms of O_γ_ and HG of S211 and the N8 atom of ceftazidime. Each point represents a snapshot from the molecular dynamics simulations, colored according to its counts shown in the color bar on the top right corner.

CMY-30 and CMY-42 are single mutants of CMY-2 harboring the substitutions of V211 to glycine and serine, respectively (17, 26). CMY-30 and CMY-42 are reported to hydrolyze oxyimino-cephalosporins such as ceftazidime more efficiently than CMY-2 with higher turnover rates. According to the studies of the molecular dynamics simulation for CMY-30 and CMY-42, the V211 substitution increases the fluctuation of the structure spanning the Ω loop to the Q120-loop, resulting in better accommodation of the substrate cephalosporin for the deacylation reaction and leading to efficient release of the product (27, 28). This increase in fluctuation is caused by the removal of restriction on the Y221 residue, which is usually restrained by V211 in CMY-2. In addition, CMY-107, which is a single mutant of CMY-2 harboring the Y199C substitution, possesses similar hydrolytic efficiency to that of CMY-30 or CMY-42, and it is also reported to display incremental fluctuation of the structure from the Ω loop to the Q120-loop due to the motile side chain of the unrestrained Y221 residue (29). We could not observe an increase in the fluctuation of the structure around the Y221 residue during the molecular dynamics simulations for the CMY-185-ceftazidime complex (Fig. 4a). In the CMY-185-ceftazidime complex, the K120 residue formed a stable salt bridge with the D123 residue. Both residues K120 and D123 are located at the Q120 loop, and the Q120 loop structure is rigidified due to this interaction, suggesting a cause for the restriction of fluctuation. In fact, the *k*_cat_ value of CMY-185 was lower than that of V211S (CMY-42) or V211S_N346Y (Table 1). However, fluctuation of the structure of the aminothiazole ring included in the R1 side chain of ceftazidime notably increased in comparison to that of the CMY-2-ceftazidime complex. Fig. 5 shows the distance distribution between the aminothiazole ring of ceftazidime and each enzyme. A single peak was observed in the CMY-2-ceftazidime complex and positioned around (0.95, 0.62) nm for the distances between the N8 atom of ceftazidime and the C_α_ atoms for E61 or Y221, respectively (Fig. 5b). On the other hand, the 2D histogram of the CMY-185-ceftazidime complex showed the broad distribution of the distances, and at least four peaks were observed with similar levels around (0.92, 0.78), (1.21, 0.90), (1.22, 1.19), and (1.25, 0.90) nm for the distances between the N8 atom of ceftazidime and the C_α_ atoms of E61 or Y221, respectively (Fig. 5c). These findings indicate that the ceftazidime molecule in the CMY-185 complex is more flexible than that in the CMY-2 complex. In addition, the O_γ_ atom of S211 formed a stable hydrogen bond with the side chain of E61 and was sandwiched between the N8 atom of ceftazidime and the side chain of E62 (Fig. 4d). Fifteen percent of the snapshots during the molecular dynamics simulation showed that the distances between the O_γ_ atom of S211 and the N8 atom of ceftazidime were within 3.5 Å (Fig. 6). Hydrogen bonds were observed in 51% of these distances within 3.5 Å (8% of overall distances). These results suggest that the interaction between S211 and N8 atom of ceftazidime through a weak hydrogen bond might contribute to stimulate the fluctuation of ceftazidime to pull the R1 side chain of ceftazidime toward the Ω loop.

The structural change of the R2 loop in CMY-185 leads to further expansion of the substrate binding site for cephalosporins, and this expansion is a major cause for the reduced susceptibility to cefiderocol of the strain. A similar expansion of the substrate binding site is observed in AmpC^Ent385^, which contains the deletion of residues A294 and P295 and also confers reduced susceptibility to cefiderocol (6, 15). In addition, CMY-172 and CMY-178 contain the deletion of three residues K290, V291, and A292 on the R2 loop, resulting in the higher MICs of 16 and 64 mg/L for ceftazidime-avibactam, respectively, and 1 mg/L for cefiderocol (30). Moreover, our results indicated that the CMY-2-like β-lactamases strongly recognize cefiderocol compared to the other β-lactamases. To our knowledge to date, the lowest *K*_m_ value against cefiderocol is 49.8 µM for AmpC^Ent385^ (15) and the *K*_m_ values for other β-lactamases are over 190 µM (19, 31). Compared to these values, CMY-2 had a notably lower *K*_i app_ value of 8.0 µM, and N346Y and A114E_N346Y showed even lower *K*_i app_ values of 0.7 and 0.4 µM, respectively. In addition, the *K*_i app_ values for N346Y and A114E_N346Y against cefiderocol were comparable to those against ceftazidime. These findings suggest that the CMY-2-like enzymes strongly recognize the structure around the R1 side chain and the cephalosporin cephem core, which is shared between ceftazidime and cefiderocol, and this may serve as the driving force for the rotation of Y346. We observed that the CMY-2 variants harboring the N346Y substitution exhibit enhanced substrate inhibition during the nitrocefin hydrolysis. The Y346 residue is required to rotate for recognition of cephalosporins; therefore, this rotation may be a rate-limiting step of the enzyme reaction for the substrates exhibiting high turnover rate such as nitrocefin. Before the substrate is accommodated, there would be a pre-binding state, where the CMY-2 variants harboring the N346Y substitution only recognize the structure around the R1 side chain of cephalosporin, resulting in the substrate inhibition. Thus, we propose the following enzyme reaction scheme: the CMY-2 variant harboring the N346Y substitution initially recognizes the structure around R1 side chain of cephalosporin, then the steric hindrance between the cephalosporin and the enzyme induces the rotation of Y346 to accommodate the substrate, and finally the activated S64 residue nucleophilically attacks the C8 atom of the cephalosporin to cleave the β-lactam ring. This hypothesis also appears to be consistent with the rejection of avibactam binding to the CMY-2 variants harboring N346Y substitution. The oxyimino group is the major R1 side chain of oxyimino-cephalosporins (32); thus, the diazabicyclooctane derivatives with substitution of 2-carbamoyl group with the oxyimino group may be potential inhibitors of mutated β-lactamases conferring resistance to valuable antibiotics like ceftazidime-avibactam or cefiderocol.

### Conclusion

Evolution of antimicrobial resistance caused by antimicrobial selection pressure is a significant threat and requires close monitoring. The CMY-185 enzyme is a CMY-2 variant possessing four amino acid substitutions A114E, Q120K, V211S, and N346Y, and was identified in an *E. coli* strain isolated from a patient who was treated with the ceftazidime-avibactam combination. CMY-185 is a unique enzyme in which the level of resistance to ceftazidime-avibactam is incrementally enhanced with the accumulation of amino acid substitutions. Here, we revealed the functional role of each substituted residue in CMY-185 using biochemical and structural analyses. The N346Y substitution confers resistance to avibactam inhibition and also induces a drastic structural change of CMY-185 with the expansion of the substrate binding site. The Q120K substitution enhances resistance to the avibactam inhibition, while the V211S substitution increases the turnover rate of the cephalosporin hydrolysis. The A114E substitution combined with the other substituted residue appears to adjust the binding affinity of the substrate. These findings demonstrate that CMY-185 has achieved an optimal balance between the turnover rate and the binding affinity for hydrolyzing ceftazidime while maintaining high-level resistance to avibactam inhibition. CMY-185 has acquired these enzymatic characteristics and is able to confer high-level resistance to ceftazidime-avibactam. The findings in this study provide new insights into how β-lactamases evolve in bacteria by accumulating beneficial substitutions to survive the selective pressure of the latest β-lactam agents.

## MATERIALS AND METHODS

### Cloning, expression, and purification of the CMY-2 variants

In this study, all CMY-2 variant genes encoding the mature enzyme without a signal peptide were cloned into the protein expression plasmid vector pET-30b between the restriction enzyme sites of NdeI and EcoRI. The expression and purification of all CMY-2 variants were performed in the same way as follows. Lysogenic broth plus kanamycin (30 µg/mL) was inoculated with *E. coli* BL21 (DE3) harboring each pET-30b-CMY-2 variant recombinant plasmid and grown at 30°C to an OD_600_ of 0.5–0.7. A final concentration of 0.1 mM IPTG was added, and the culture was incubated for an additional 3 h. The cells were harvested by centrifugation at 3,500 × *g* for 10 min at 15°C, and the pellet was resuspended in 20 mM Tris-HCl pH 7.0 buffer, sonicated, and centrifuged at 8,000 × *g* for 30 min at 4°C. The supernatant was transferred to the new centrifuge tube and further centrifuged at 48,000 × *g* for 90 min at 4°C. The crude extract was loaded onto a HiTrap SP HP (Cytiva, Tokyo, Japan) previously equilibrated with 20 mM Tris-HCl pH 7.0 buffer. The CMY-2 variant was then eluted with a NaCl linear gradient of 0–0.5 M. The peak fractions containing the CMY-2 variant were pooled and then dialyzed overnight with 20 mM Tris-HCl pH 7.0 buffer. Further purification was performed with a HiTrap Blue HP column (Cytiva, Tokyo, Japan) chromatography, and the CMY-2 variant was eluted with a linear gradient of 0–2 M NaCl in 20 mM Tris-HCl pH 7.0 buffer. The active fractions were confirmed with BD BBL Cefinase Paper Disc (Becton Dickinson, Sunnyvale, CA, USA), pooled and concentrated using a Vivaspin turbo 4 centrifugal concentrator (MWCO 10,000, Sartorius, Gottingen, Germany). The buffer was exchanged to 20 mM Tris-HCl pH 7.0 by several rounds of dilution and concentration. The purified active CMY-2 variant solution was finally concentrated to 10–20 mg/mL and stored in 20 µL aliquots at −80°C until used in crystallization and kinetics experiments.

### Kinetic measurements

The steady-state kinetics were carried out on a UV-Visible spectrometer (UV-1280, Shimadzu, Japan) in PBS pH 7.2 at 25°C with a constant amount of enzyme and varying concentrations of the substrates. We tested nitrocefin, cephalothin, ceftazidime, and cefiderocol as the substrates and avibactam as the inhibitor. The steady-state kinetics parameters *K*_m_ and *k*_cat_ were determined with the Michaelis-Menten equation. For poorly hydrolyzed substrates, the *K*_m_ value was assumed to be the competitive inhibition constant (*K*_i app_) value according to previous studies (17, 19). Inhibition parameter values of *K*_i app_, *k*_2_/*K*, *k*_−2_, and *k*_off_ for avibactam were determined using a method as described previously (33). Cephalothin was used as the reporter substrate for competitive inhibition experiments, and its concentration was fixed at 100 µM. The enzyme concentration was 1–10 nM. Inverse steady-state initial velocities were plotted against the concentration of the competitive inhibitor agents, such as ceftazidime, cefiderocol, and avibactam. The data were analyzed by linear regression analysis of the Dixon plot, and *K*_i app (observed)_ was determined by dividing the *y*-intercept value by the slope of the line. It was then corrected to account for the affinity of cephalothin for each enzyme using the Equation (1):


(1) $$K_{i\;app}=K_{i\;app\;(observed)}/\left\{1+[S]/K_{m\;(cephalothin)}\right\}$$

where [S] represents the concentration of the reporter substrate cephalothin, and *K*_m (cephalothin)_ is the *K*_m_ value of cephalothin for each enzyme.

The *k*_off_ value was determined using a jump dilution assay. The enzyme and avibactam were mixed and preincubated at 25°C for 5 min. The concentration of avibactam depended on the enzyme and concentrations 10-fold higher from each *K*_i app (observed)_ value were adopted, which were determined based on the corrected competitive inhibition constants *K*_i app_. Preincubated enzyme-avibactam mixture was then diluted 2,000-fold in the 100 µM cephalothin solution, and the hydrolysis of cephalothin was measured. Three independent measurements were performed for each enzyme and substrate or inhibitor combination. Curve fittings using linear or non-linear regression were performed with R 4.1.3 (R Core Team, 2022).

### Crystallization of CMY-185

Prior to the crystallization, the purified CMY-185 recombinant enzyme was diluted to the final concentration of 15 mg/mL in 20 mM Tris-HCl pH 7.0. Crystals of CMY-185 were obtained using hanging-drop vapor diffusion method with the crystallization condition in which the CMY-185 solution was mixed with an equal volume of a reservoir solution containing 24% PEG 20,000, 0.1 M Tris-HCl pH 8.5, and 0.2 M lithium sulfate at 20°C. Crystals of the CMY-185-ceftazidime complex were obtained by soaking the CMY-185 crystals in a solution containing 100 mM ceftazidime, 26% PEG 20,000, 0.1 M Tris-HCl pH 7.0, and 0.2 M lithium sulfate at 4°C for 4 h.

### Data collection, structure determination, and refinement

The CMY-185 crystals were transferred into a cryoprotectant solution composed of reservoir solution containing 15% glycerol, and then flash-frozen in liquid nitrogen. Synchrotron experiments were performed at Photon Factory BL-17A (High Energy Accelerator Research Organization, Tsukuba, Japan). Diffraction data sets were collected at −173°C using an EIGER X16M detector, and were processed and scaled using *XDS* (34). The initial phase of the CMY-185 structure was determined by the molecular replacement method using *Molrep* (35) from the *CCP4* program suite (36), with the coordinate (PDB ID: 6G9T) serving as the search model (24). Manual model rebuilding was performed with *COOT* (37). Structure refinements were performed with *phenix.refine* from the *PHENIX* package (38). The CMY-185 free-form structure was refined with the anisotropic atomic displacement parameters for the heavy atoms of protein. The CMY-185-ceftazidime complex structure was refined with the atomic displacement parameters using the translation, liberation, and screw (TLS) method, and the TLS groups were determined by using *phenix.find_tls_groups*. The stereochemical quality of the final structures were evaluated by *MolProbity* (39). All molecular graphics were prepared using *PyMOL* (Schrödinger, L. & DeLano, W., 2020. *PyMOL*, Available at: http://www.pymol.org/pymol.).

### Molecular dynamics simulations

Molecular dynamics simulations were run for the free forms and the ceftazidime complexes with CMY-2 and CMY-185 using *GROMACS* (40) and the ff14SB forcefield (41). Prior to the simulation, the loop and the side chain structures disordered in the crystal structure of the CMY-185-ceftazidime complex were modeled using *MODELLER* (42). The model structure of the CMY-2-ceftazidime complex was generated by superimposing the structures of the CMY-185-ceftazidime complex on CMY-2 (PDB ID 1ZC2). The protonation state of each residue was predicted with *PROPKA 3* (43). During the deacylation reaction in the AmpC β-lactamases, Y150 is assumed to be deprotonated and acts as a general base in activating hydrolyzing water molecule (44), therefore the Y150 residue instead of the K67 residue in the model structure of each complex was deprotonated. The parameters for the residues S64 acylated with ceftazidime and deprotonated Y150 were generated with the GAFF forcefield (45) and AM1-BCC partial charges (46) using *Antechamber* (47) from the *AmberTools 20* software (48). After adding hydrogen atoms, the modeled structure of each CMY-2 and CMY-185 was placed in a cubic box with a boundary of 10 Å under a periodic boundary condition. The system was solvated with TIP3P water, and then neutralized by adding sodium and chloride ions using *LEaP* (49). Amber topology files were converted to GROMACS topology files using *ACPYPE* (50). The energy minimization of the system was performed with the steepest descent algorithm, and then the system was heated gradually from 0 to 300 K in NVT ensemble for 200 ps with harmonic positional restraints on the heavy atoms of protein and ligand (force constant, 10 kcal/mol/Å^2^). The system was relaxed at 300 K in NPT ensemble for 100 ps with the positional restraints maintained, and the force constants of the positional restraints were gradually reduced to 0 kcal/mol/Å^2^ for further 700 ps. The equilibrated system was then subjected to molecular dynamics simulation for 100 ns. All bonds were constrained with the LINCS algorithm (51). Cutoffs of 1.0 nm were used for the neighbor list, Coulomb interactions and Van der Waals interactions. Long-range electrostatic interactions were measured using the particle-mesh Ewald algorithm (52). The time step of the molecular dynamics simulation was set to 2 fs, and the snapshots were stored every 10 ps. The simulations were performed with five runs for each system and all data were subjected to analysis.

## Data Availability

The atomic coordinates of the CMY-185 free form and the CMY-185-ceftazidime complex have been submitted to the Protein Data Bank under PDB accession numbers 8JB7 and 8JB8, respectively.

## SUPPLEMENTAL MATERIAL

Supplemental figures mbio.02874-23-s0001.pdf Fig. S1 to S4.

ASM does not own the copyrights to Supplemental Material that may be linked to, or accessed through, an article. The authors have granted ASM a non-exclusive, world-wide license to publish the Supplemental Material files. Please contact the corresponding author directly for reuse.
