# Supplementary material for: Structural insights into the molecular mechanism of high-level ceftazidime–avibactam resistance conferred by CMY-185
Source: mBio. 2024 Jan 5;15(2):e02874-23. doi: 10.1128/mbio.02874-23 (PMC10865806; doi:10.1128/mbio.02874-23)
Supplement: Supplemental figures — Fig. S1 to S4. [file mbio.02874-23-s0001.pdf]

Supplemental Material

Structural insights into the molecular mechanism of high-level ceftazidime–avibactam resistance conferred by CMY-185

Akito Kawai,[1,2] William C. Shropshire,[3] Masahiro Suzuki,[1,2] Jovan Borjan,[4] Samuel L. Aitken,[4] William C. Bachman,[5] Christi L. McElheny,[5] Micah M. Bhatti,[6] Ryan K. Shields,[5] Samuel A. Shelburne,[3] Yohei Doi[1,2,5,7]

[1]Department of Microbiology, Fujita Health University School of Medicine, Toyoake, Aichi, Japan

[2]Center for Infectious Disease Research, Fujita Health University, Toyoake, Aichi, Japan

[3]Department of Infectious Diseases, Infection Control, and Employee Health, The University of Texas MD Anderson Cancer Center, Houston, TX

[4]Division of Pharmacy, The University of Texas MD Anderson Cancer Center, Houston, TX

[5]Division of Infectious Diseases, University of Pittsburgh School of Medicine, Pittsburgh, PA

[6]Department of Laboratory Medicine, Division of Pathology/Lab Medicine, The University of Texas MD Anderson Cancer Center, Houston, TX

[7]Department of Infectious Diseases, Fujita Health University School of Medicine, Toyoake, Aichi, Japan

Corresponding authors:

Akito Kawai, PhD, 1-98, Dengakugakubo, Kutsukake-cho, Toyoake, Aichi, 470-1192, Japan, phone: +81-562-93-2433, fax: +81-562-93-4003, email: kawai-a@fujita-hu.ac.jp

Yohei Doi, MD, PhD, BST E1057, 200 Lothrop Street, Pittsburgh, PA 15261, United States, phone: 412-648-9445, fax: 412-648-8521, email: yod4@pitt.edu

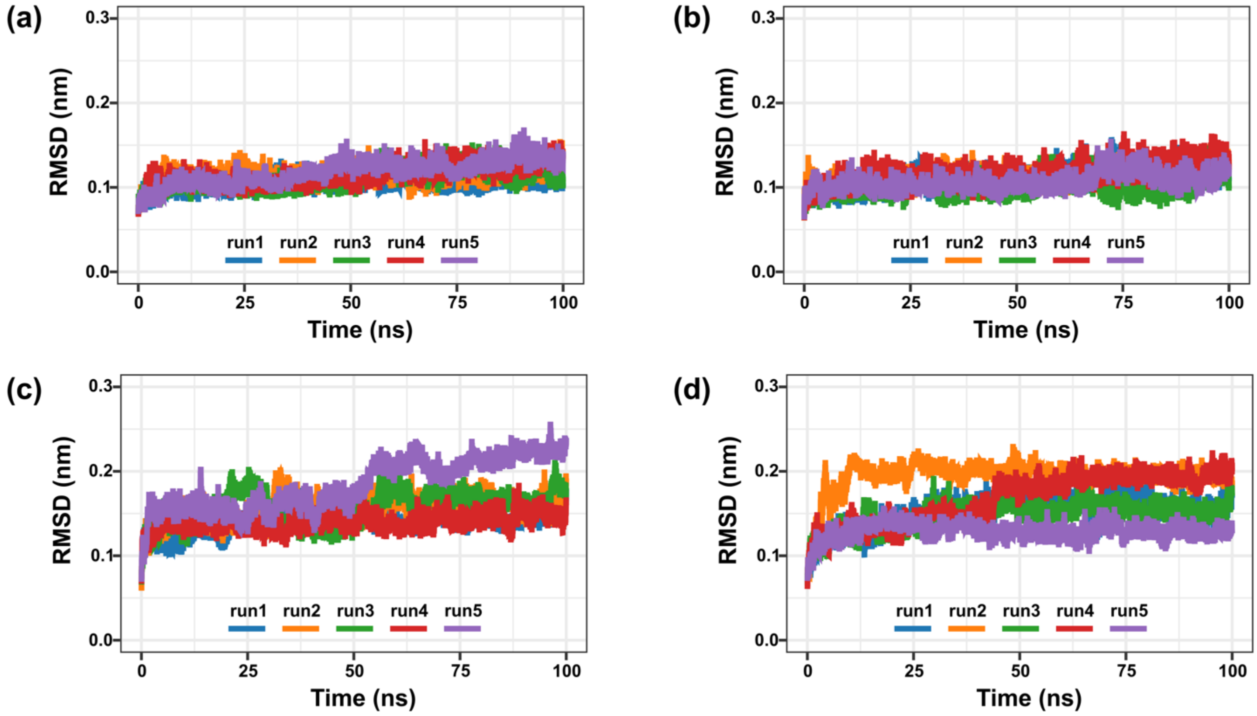

Figure S1. The RMSD profile from the intial structure of the molecular dynamics simulation. The systems of CMY-2 in the free form (a) and the acyl complex with ceftazidime (b). The systems of CMY-185 in the free form (c) and the acyl complex with ceftazidime (d).

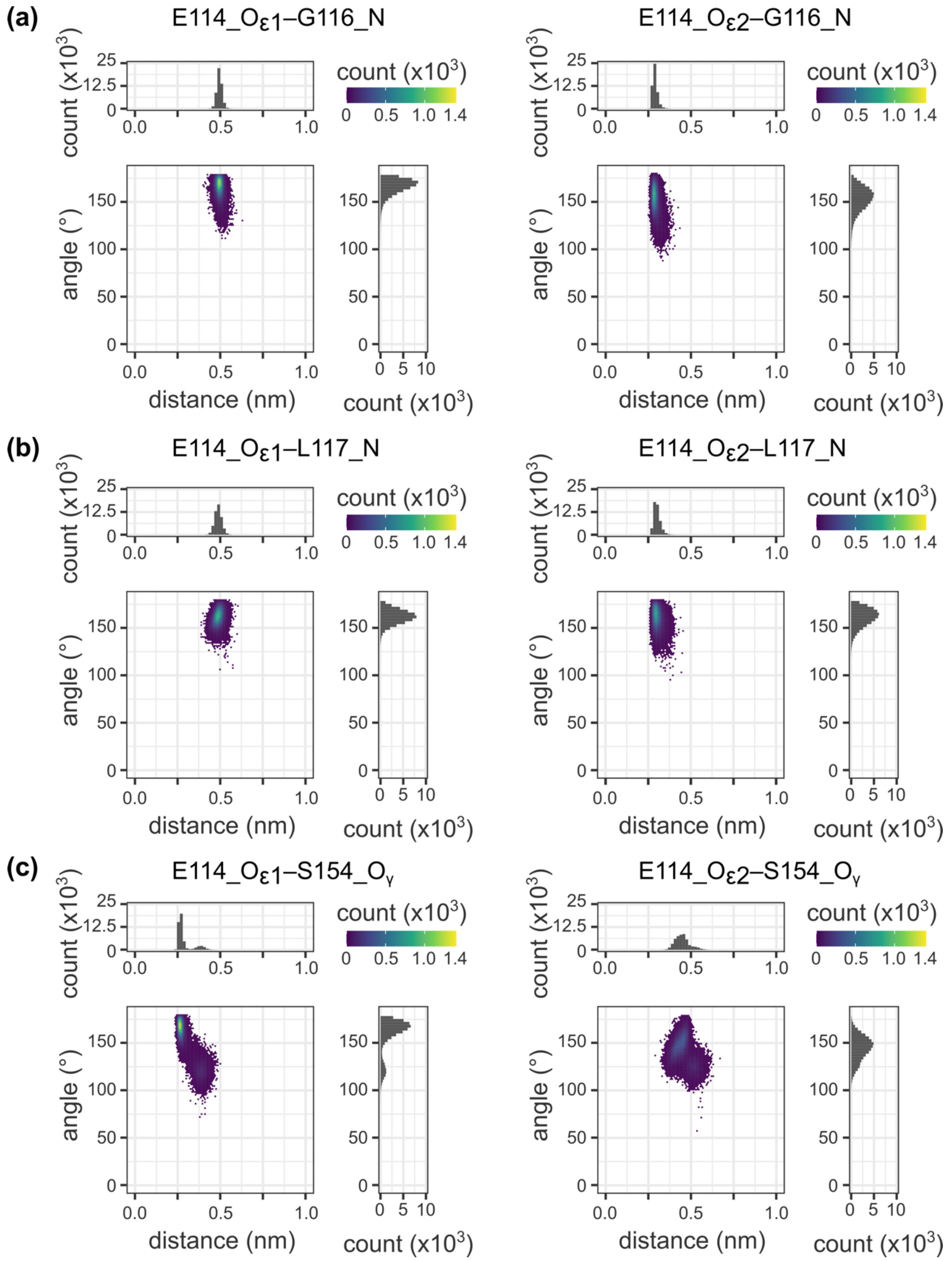

Figure S2. Hydrogen bonds between E114 and G116, L117 or S154.

2D histogram for the distribution for the distance and angle. “Distance” axis indicates the distance between the $O_{\varepsilon1}$ atom (left) or the $O_{\varepsilon2}$ atom (right) of E114 and the main chain N atom of G116 (a), L117 (b) or the O_{γ} atom of S154 (c). “Angle” axis indicates the angle between the central hydrogen atom and each heavy atom. Each points represents a snapshot from the molecular dynamics simulations, colored according to its counts shown in the color-bar on the top right corner.

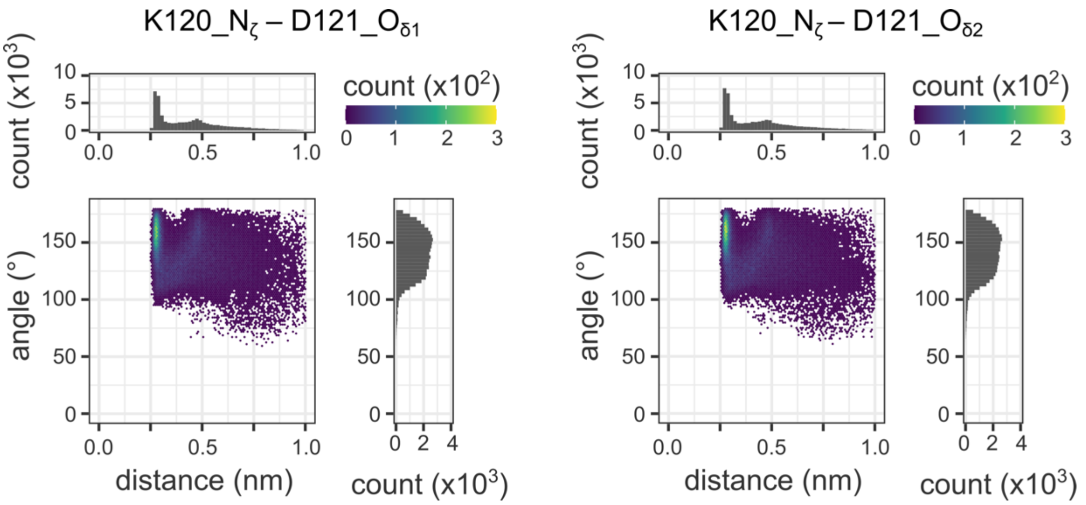

Figure S3. Hydrogen bonds between K120 and D121.
2D histogram for the distribution for the distance and angle. "Distance" axis indicates the distance between the $N_ζ$ atom of K120 and the $O_{δ1}$ atom (left) or the $O_{δ2}$ atom (right) of D121. "Angle" axis indicates the angle between the central hydrogen atom and each heavy atom. Each points represents a snapshot from the molecular dynamics simulations, colored according to its counts shown in the color-bar on the top right corner.

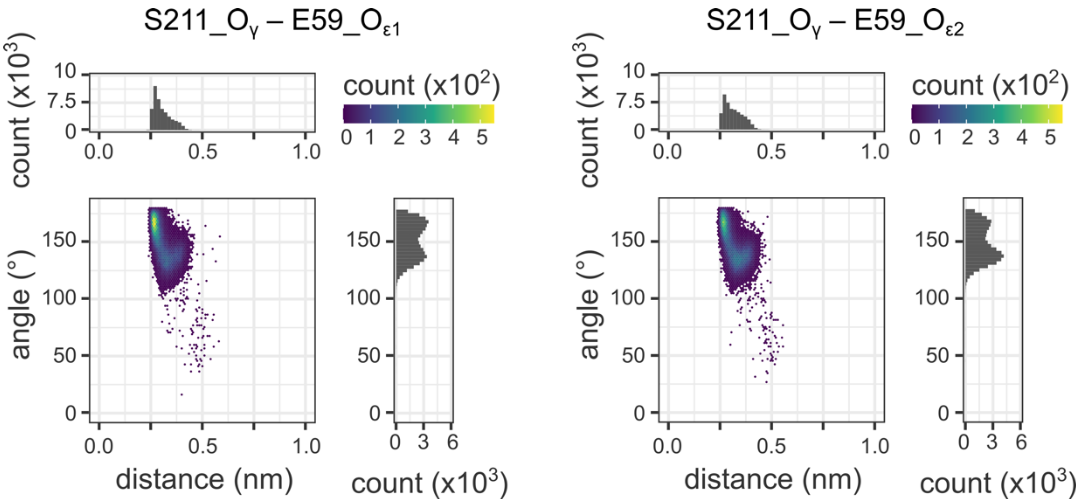

Figure S4. Hydrogen bonds between S211 and E59.

2D histogram for the distribution for the distance and angle. “Distance” axis indicates the distance between the O_γ atom of S211 and he $O_{\varepsilon 1}$ atom (left) or the $O_{\varepsilon 2}$ atom (right) of E59. “Angle” axis indicates the angle between the central hydrogen atom and each heavy atom. Each points represents a snapshot from the molecular dynamics simulations, colored according to its counts shown in the color-bar on the top right corner.
